# Chromosome-scale genome assembly of the fire blight resistant *Malus fusca* accession MAL0045, donor of *FB_Mfu10*

**DOI:** 10.1038/s41597-025-05232-0

**Published:** 2025-05-27

**Authors:** Ofere Francis Emeriewen, Thomas Wolfgang Wöhner, Henryk Flachowsky, Andreas Peil

**Affiliations:** Julius Kühn-Institut (JKI) – Federal Research Centre for Cultivated Plants, Institute for Breeding Research on Fruit Crops, Dresden-Pillnitz, Germany

**Keywords:** Plant sciences, Genetics

## Abstract

The wild apple, *Malus fusca* accession MAL0045, is highly resistant to fire blight disease, caused by the bacterial pathogen, *Erwinia amylovora*. A major resistance locus, *FB_Mfu10* was identified on chromosome 10 of MAL0045 including other contributory loci on chromosomes 16, 4, and 15. Here, we report a chromosome-scale genome assembly of MAL0045 to facilitate the studies of its fire blight resistance. PacBio sequencing and Illumina sequencing for Hi-C contig anchorage were employed to obtain the genome. A total of 669.46 Mb sequences were anchored onto 17 chromosomes, taking up 99.75% of total contig length. Contigs anchored onto chromosomes were further ordered and orientated, where a total of 637.67 Mb sequences were anchored onto chromosomes in proper order and orientation, resulting in a final anchoring ratio of 95.25%. The BUSCO score of this assembly is 97.46%. Further, a total of 47,388 genes were predicted via *ab initio*, homology-based, and RNAseq methodologies. The availability of this genome will facilitate functional and comparative genomics studies, especially about the donors of fire blight resistance in *Malus*.

## Background & Summary

The genus *Malus* Mill. of the Rosaceae family comprises the domesticated apple (*Malus domestica* Borkh.) and its wild relatives^1,2^. Members of this genus, especially the domesticated apple, are highly susceptible to fire blight, which is the most destructive bacterial disease, caused by the gram-negative bacterium *Erwinia amylovora*^3^ that plague the genus. However, some wild *Malus* genotypes are resistant and are donors of fire blight resistance loci and genes that have been identified and mapped to the apple genome^4,5^. One such wild genotype is the Oregon crab apple, *Malus fusca* accession MAL0045 of the genebank of the Julius Kühn Institute in Germany, which of all the *M. fusca* accessions, was found to be the most resistant to *E. amylovora* following artificial shoot inoculations with different strains of the bacterium^6–8^. Following chromosome walking studies involving the development of tightly linked markers using published apple genome sequences^9,10^, and sequencing of bacterial artificial chromosome (BAC) clones that span the region of resistance, the locus, *FB_Mfu10*, was precisely mapped using 1,888 F_1_ individuals and candidate genes were proposed^11,12^.

Furthermore, due to the strong resistance of MAL0045 to highly virulent North American strains of the pathogen e.g. Ea3049, but a large ratio of susceptibility in its F_1_ progeny to Ea3049, it was hypothesized that homozygous loci are present in the MAL0045 genome contributing to its fire blight resistance, which were not detected in the F_1_ progeny. Two resistance loci regions on chromosome 4 and 15 of MAL0045, not detected in the F_1_ progeny, were subsequently identified using F_2_ progeny that contribute to the resistance of MAL0045^13^. The *FB_Mfu10* locus and associated candidate genes were analysed using the recently published genome of another *M. fusca* accession, PI 589975, of the United States Department of Agriculture (USDA) Plant Genetic Resources Unit (PGRU) *Malus* collection. It was therein hypothesized by the authors of the genome of PI 589975^14^ that copy number variation (CNV) of the G-type lectin S-receptor-like serine/threonine protein kinase genes, which were previously proposed^11^, potentially contributes to the resistance of *M. fusca*. However, it is important to note that PI 589975 is not the donor of *FB_Mfu10*, and there is variability of fire blight resistance and susceptibility even amongst different accessions of the same genotype. Therefore, it is imperative to sequence and annotate the genome of MAL0045 and make it available to the scientific community.

Here, we report a chromosome-scale genome sequence using PacBio sequel II platform and Hi-C anchoring of MAL0045 (MfuMAL45).

## Methods

### Plant material and DNA isolation

Young leaves and buds of *Malus fusca* accession MAL0045 were harvested from the orchard in spring, immediately frozen in liquid nitrogen, and thereafter stored in −80 °C until required for DNA extraction. High-quality genomic DNA was extracted from leaves using a modified CTAB method according to Almakarem *et al*.^15^. RNaseA was used to remove RNA contaminants. The quality of the DNA was checked by agarose gel electrophoresis before use in further analyses.

### Genome survey, PacBio HiFi (CCS) library construction and sequencing

A short-read library of 350 bp was constructed for genome survey sequencing. Sequencing was performed on an Illumina NovaSeq platform (Illumina, CA, USA), resulting in 32.83 Gb of clean reads.

High molecular weight DNA was sheared into ~ 15 kb fragments using Megaruptor® 2, and SMRTbell library was constructed using the SMRTbell Express Template Prep kit 3.0 (Pacific Biosciences). Briefly, first enzymatic reaction removed single-stranded overhangs from 10 µg of the sheared DNA, which was subsequently treated with repair enzymes. Subsequently, ends of the double-stranded fragments were polished and tailed with an A-overhang at the 3′end. Ligation with T-overhang SMRTbell adapters was performed at 20 °C for 60 minutes. Following ligation, the SMRTbell library was digested by exonuclease and purified with 0.45X AMPure PB beads. The size distribution and concentration of the library were assessed using the FEMTO Pulse automated pulsed-field capillary electrophoresis instrument (Agilent Technologies, Wilmington, DE) and the Qubit 3.0 Fluorometer (Life Technologies, Carlsbad, CA, USA), respectively. Following library characterization, 3 µg was subjected to a size selection step using the Sage ELF system (Sage Science, Beverly, MA) to collect SMRTbells 15–18 kb. After size selection, the library was purified with 1X AMPure PB beads. Library size and quantity were assessed using the FEMTO Pulse and the Qubit dsDNA HS reagents Assay kit. Sequencing primer and Sequel II DNA Polymerase were annealed and bound, respectively, to the final SMRTbell library. The library was loaded at an on-plate concentration of 55 pM using diffusion loading. SMRT sequencing was performed using a single 8 M SMRT Cell on the Sequel II System with Sequel II Sequencing Kit, 1800-minute movies by Pacific Biosciences (USA). Low-quality reads and sequence adapters were filtered out leading to 28.80 Gb of CCS data. This is a sequencing depth of 49 X with an N50 value of 15.54 kb, and average read length of 15.06 Kb (Table 1). The distribution of read length is summarized in Table 2.

**Table 1:** Summary of PacBio HiFi sequencing.

Data_Type	Reads_num	Reads_base	Reads_LenN50	Reads_LenMean	Reads_LenMax
CCS	1,913,134	28,804,521,568	15,542	15,056	50,161

Reads_num: Counts of reads; Reads_bases(bp): Total base count; Reads_LenN50(bp): Sequence length of the shortest reads at 50% of total bases; Reads_LenMean(bp): Average read length; Reads_LenMax(bp): Longest read length. bp = base pair.

**Table 2:** Read length distribution.

Length	ReadsNumber	TotalLength	AverLength
500~2000	1,152	2,059,514	1787.77
2000~4000	8,171	26,752,199	3274.04
4000~6000	13,189	65,837,520	4991.85
6000~8000	12,924	90,657,221	7014.64
8000~10000	46,128	434,465,943	9418.70
10000~12000	330,755	3,685,290,286	11142.05
12000~14000	434,821	5,651,084,250	12996.34
14000~16000	379,884	5,682,619,946	14958.82
16000~18000	278,841	4,723,254,817	16938.88
18000~	407,269	8,442,499,872	20729.54

Note: Length: Read length range; ReadsNumber: Number of reads within corresponding read length range; TotalLength: Total length of reads within corresponding length range; AverLength: Average length of reads within corresponding length range.

We used short reads that were generated from the Illumina platform for the estimation of the genome size, the level of heterozygosity and repeat content of the genome. Long reads from the PacBio platform were used for genome assembly.

### Genome assembly by CCS data and features estimation from K-mer

**Fig. 1:**
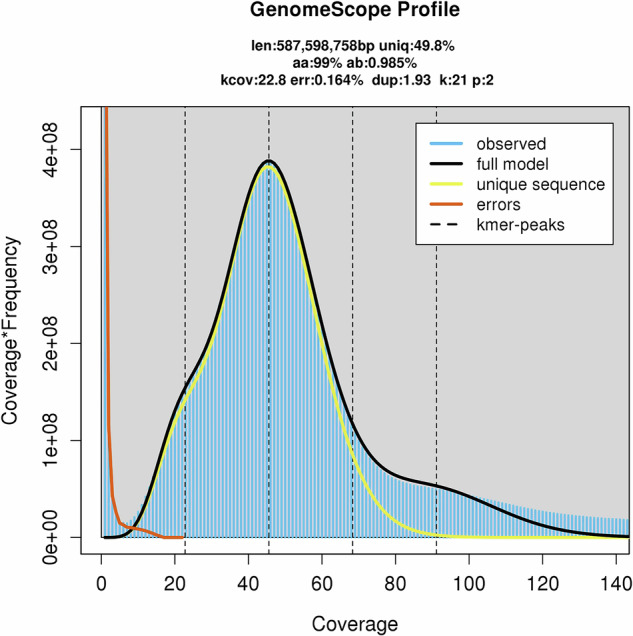
Frequency distribution of coverage and K-mer numbers.

We assembled the 28.80 Gb of CCS data using Hifiasm (v 0.16) software^16^ resulting in a genome containing 575 contigs. Short-reads from the Illumina platform were quality filtered by fastp^17^ using the parameters -q 10 -u 50 -y -g -Y 10 -e 20 -l 100 -b 150 -B 150. We counted the 21-kmers with Jellyfish software^18^ and calculated the genome characteristics using Genomescope software. The genome size of MfuMAL45 was estimated to be 587.60 Mb. The heterozygosity of the genome was 0.98%. A k-mer distribution map with k = 21 is shown in Fig. 1.

### Hi-C sequencing and Contigs anchoring

**Fig. 2:**
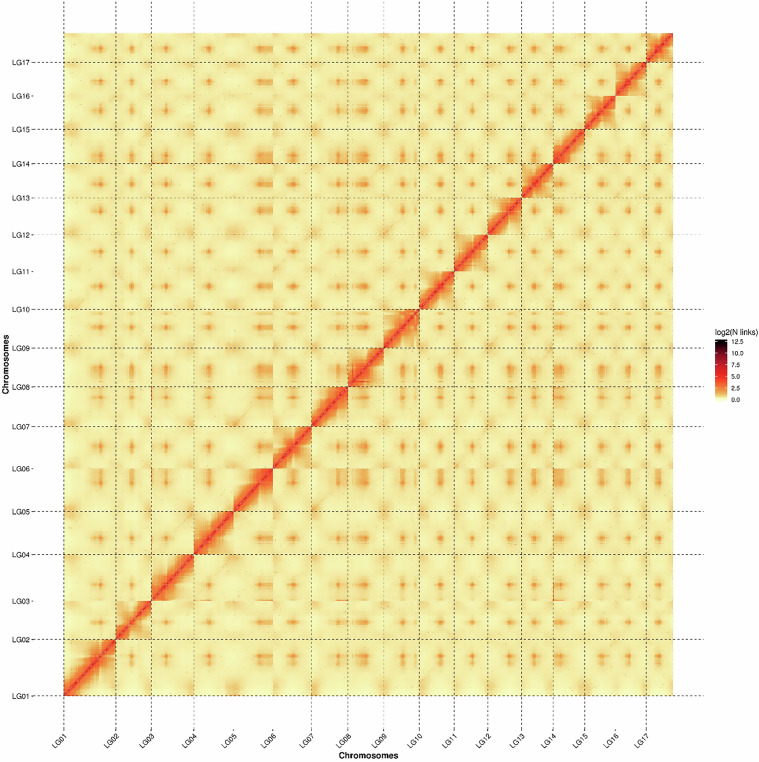
Hi-C interaction heatmap between the assembled 17 chromosomes of *Malus fusca* MfuMAL45 genome.

For Hi-C based contig anchoring, a fragment library from 300–700 bp insert size was constructed and sequenced through Illumina NovaSeq platform resulting in 70.77 Gb clean data following the removal of adapter sequences and low quality reads. We mapped the 70.77 Gb to the MfuMAL45 genome using BWA (bwa-0.7.17) with the default parameters. We successfully clustered 548 contigs into 17 groups with the agglomerative hierarchical clustering method in Lachesis^19^. In addition, clustered contigs were ordered using Lachesis^19^. Subsequently, 669.46 Mb sequences were anchored onto 17 chromosomes, which took up 99.75% of the total contig length (Table 3). We obtained the chromosomal-level high-quality assembly in anchored sequences of 637.67 Mb in confirmed order and orientation, which took up 95.25% of the total sequence length. Statistics on Hi-C contigs assembly is shown in Table 3. Hi-C interaction heatmap within the chromosomes is shown in Fig. 2.

**Table 3:** Statistics of Hi-C contig anchorage.

Linkage Group	Cluster length	Order length
LG1	54,374,751	54,301,286
LG2	46,601,243	36,912,530
LG3	44,894,749	44,691,075
LG4	41,986,527	41,652,006
LG5	46,350,876	41,110,250
LG6	40,583,972	40,251,138
LG7	38,508,174	38,338,780
LG8	37,398,228	37,350,734
LG9	37,340,033	37,234,748
LG10	36,572,406	36,489,418
LG11	35,784,901	35,302,691
LG12	35,696,903	35,641,590
LG13	47,364,435	32,713,194
LG14	33,296,939	33,196,904
LG15	32,289,064	32,251,003
LG16	32,215,895	32,137,758
LG17	28,205,526	28,093,631
Total	669,464,622 (99.75%)	637,668,736 (95.25%)

### Annotation of repetitive sequences

The following workflows annotated transposon element (TE) and tandem repeat. We identified TEs by a combination of homology-based and *de novo* approaches. Firstly, we used RepeatModeler (http://www.repeatmasker.org/RepeatModeler/)^20^ to customize a *de novo* repeat library of the MfuMAL45 genome. Subsequently, full-length long terminal repeat retrotransposons (fl-LTR-RTs) were identified with LTRharvest (v1.5.9)^21^ and LTR_finder (v2.8)^22^. The fl-LTR-RTs and LTR library were thereafter produced by LTR_retriever^23^. A non-redundant species-specific TE library was constructed by combining the *de novo* TE sequences library using the Dfam (v3.5) database. Finally, TE sequences in the MfuMAL45 genome was identified and grouped by homology-based search in the library using RepeatMasker (v4.12)^24^. Tandem repeats were annotated with Tandem Repeats Finder (TRF v4.09)^25^ and the MIcroSAtellite Identification Tool (MISA v2.1)^26^. A total of 366,217,460 bp TE sequence was identified, which resulted in 54.57% of the entire genome. Detailed information on TE prediction is provided in Table 4.

**Table 4:** Statistics of TE sequences.

Type	Number	Length	Rate (%)
ClassI:Retroelement	426,652	301,667,109	44.95
ClassI/LINE	29,724	8,256,975	1.23
ClassI/LTR/Caulimovirus	1,111	1,440,768	0.21
ClassI/LTR/Copia	85,525	83,190,565	12.40
ClassI/LTR/ERV	3,999	326,071	0.05
ClassI/LTR/Gypsy	83,581	106,889,613	15.93
ClassI/LTR/Ngaro	193	12,961	0.00
ClassI/LTR/Pao	2,082	478,032	0.07
ClassI/LTR/Unknown	201,069	97,371,492	14.51
ClassI/SINE	19,368	3,700,632	0.55
ClassII:DNA transposon	256,687	64,548,664	9.62
ClassII/Academ	2	80	0.00
ClassII/CACTA	2,385	1,399,836	0.21
ClassII/Crypton	17	621	0.00
ClassII/Dada	207	11,234	0.00
ClassII/Ginger	22	1,252	0.00
ClassII/Helitron	130,406	33,638,365	5.01
ClassII/IS3EU	146	8,073	0.00
ClassII/Kolobok	209	20,363	0.00
ClassII/Maverick	103	6,149	0.00
ClassII/Merlin	90	4,685	0.00
ClassII/Mutator	466	31,622	0.00
ClassII/P	59	2,893	0.00
ClassII/PIF-Harbinger	4,508	2,124,297	0.32
ClassII/PiggyBac	28	1,331	0.00
ClassII/Tc1-Mariner	210	12,138	0.00
ClassII/Unknown	112,827	24,736,739	3.69
ClassII/Zisupton	88	3,978	0.00
ClassII/hAT	4,914	2,545,008	0.38
Unknown	31	1,687	0.00
Total	683,370	366,217,460	54.57

### Annotation of protein coding genes

**Fig. 3:**
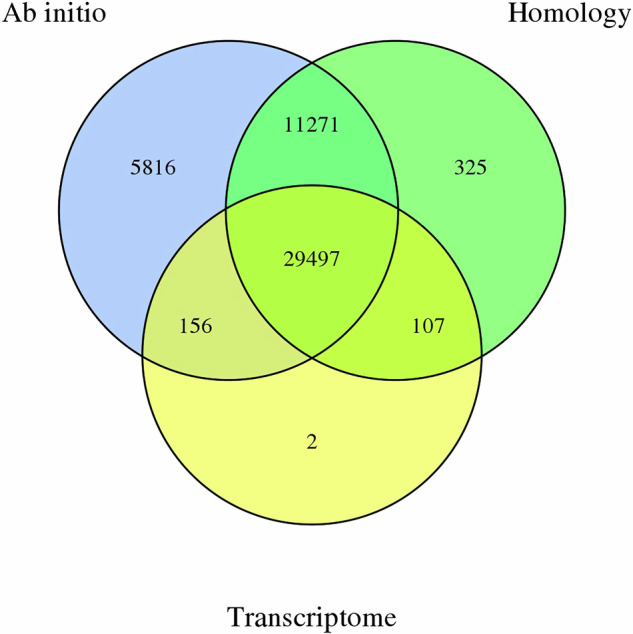
Predicted protein-coding genes from the assembled MfuMAL45 genome following *ab initio* (blue), homology-based (green) and transcriptome-based (yellow) analyses. Figure also shows genes the number of genes that were predicted in common by respective analysis methods.

We integrated three approaches, namely, *de novo* prediction, homology search, and transcript-based assembly, to annotate protein-coding genes in the genome. The de novo gene models were predicted using two *ab initio* gene-prediction software tools, Augustus (v3.1.0)^27^ and SNAP(2006-07-28). For the homolog-based approach, GeMoMa (v1.7) software^28^ was performed by using reference gene models from the other 4 species/genome versions (*Arabidopsis thaliana* v10, *Malus domestica* GDDH13/ HFTH1/Mfusca_hap1/hap2). For the transcript-based prediction, RNA-sequencing data was mapped to the reference genome using Hisat (v2.1.0)^29^ and assembled using Stringtie (v2.1.4)^30^. GeneMarkS-T (v5.1) was used to predict genes based on the assembled transcripts. We used PASA (v2.4.1) software to predict genes based on the unigenes. Full-length transcripts from the PacBio were assembled using Trinity (v2.11)^31^. The gene models identified from both approaches were combined using the EVM software (v1.1.1) and updated by PASA. In total, 47,174 protein-coding genes were predicted in the assembled MfuMAL45 genome using the criteria above (Fig. 3).

### Functional annotation of protein coding genes

**Fig. 4:**
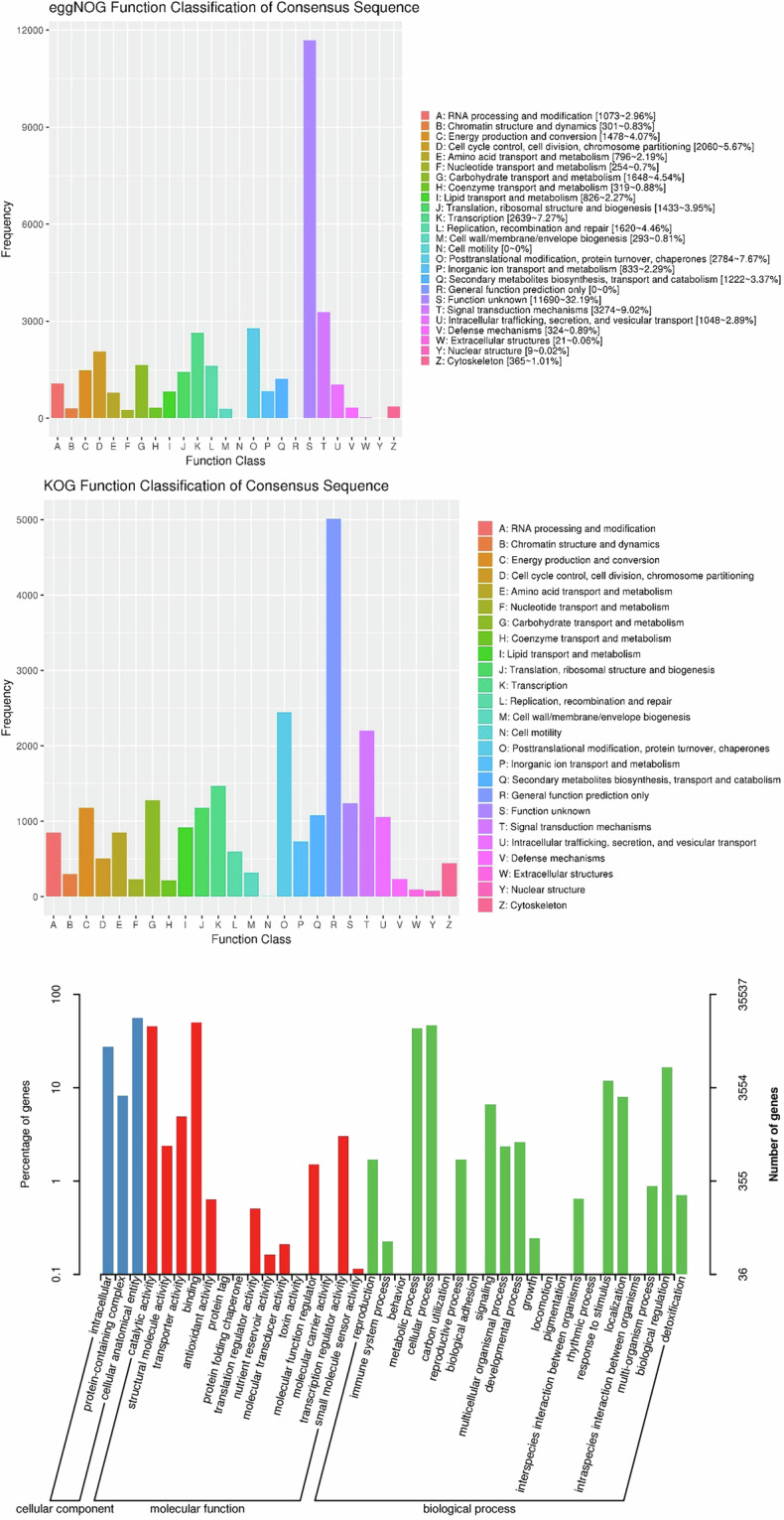
Functional annotation of predicted protein-coding genes from the assembled MfuMAL45 genome using EggNOG (**a**) up, KOG (**b**) middle, and GO (**c**) down.

We determined gene functions by aligning protein sequences to the National Center for Biotechnology Information (NCBI) Non-Redundant (NR), EggNOG^32^, KOG, TrEMBL^33^, InterPro^34^ and Swiss-Prot^33^ protein databases using diamond blastp (diamond v2.0.4.142) and the Kyoto Encyclopedia of Genes and Genomes (KEGG) database^35^. The protein domains were annotated using InterProScan (v5.34–73.0)^36^. The PFAM database^37^ was used to identify motifs and gene domains. Gene Ontology (GO) IDs for each gene were obtained from TrEMBL, InterPro and EggNOG. In total, approximately 46,440 (about 98.44%) of the predicted protein-coding genes of MfuMAL45 could be functionally annotated with known genes, conserved domains, and Gene Ontology terms, shown in Fig. 4 and Table 5.

**Table 5:** Statistics of Gene function annotation.

Anno_Database	Annotated_Number	Annotated_Ratio
GO_Annotation	35,537	75.33
KEGG_Annotation	30,378	64.4
KOG_Annotation	21,818	46.25
Pfam_Annotation	34,121	72.33
Swissprot_Annotation	30,910	65.52
TrEMBL_Annotation	46,363	98.28
eggNOG_Annotation	36,310	76.97
nr_Annotation	45,540	96.54
All_Annotated	46,440	98.44

### Annotation of Non-coding RNA genes

We used tRNAscan-SE (v1.3.1)^38^ algorithms with default parameters to identify the genes associated with tRNA. For rRNA identification, we used barrnap (v0.9) with default parameters to identify the genes associated with rRNA. MiRNAs and snRNAs were identified by Infernal (v1.1)^39^ software against the Rfam (v14.5)^37^ database with default parameters. A total of 3,476 tRNA, 7,845 rRNA, 123 miRNA, 109 snRNA and 104 snoRNA were identified.

### Pseudogene prediction

Pseudogenes were identified by scanning the genome using GenBlastA (v1.0.4) program^40^ following the prediction of functional genes. Putative candidates were then analyzed by searching for non-mature mutations and frame-shift mutations using GeneWise (v2.4.1)^41^ leading to the identification of 262 pseudogenes with a total length of 808, 235 bp and an average length of 3,084.87 bp.

## Data Records

The raw data are deposited in the European Nucleotide Archive (ENA) database under study accession PRJEB77885^42^ with accession numbers of Illumina DNA short reads (ERR14104108), RNA (ERR14104109), Hi-C short reads (ERR14104110) and PacBio reads (ERR14104112). The final assembly, repetitive sequences, structural and functional gene annotations are available from figshare^43^ and the European Nucleotide Archive (ENA)^44^.

### Technical validation (genome quality evaluation)

The assembled genome was also subjected to BUSCO v5.2.2 with the OrthoDB to evaluate the completeness of the genome^45^. BUSCO score of this assembly is 97.46%. Overall, 98.08% complete and 0.74% partial of the BUSCOs were identified in the assembled genome. In addition, we aligned short reads from the Illumina platform to the genome, which resulted in a high alignment ratio that demonstrated the high quality of contig assembly. The Cegma (v2.5) was used to evaluate the integrity of the final genome assembly. We also used bwa to align short reads (Illumina) with current genome assembly to assess the completeness and distribution of these reads on current assembly based on mapping ratio, genome coverage and sequencing depth distribution. The statistics is shown in Table 6. Minimap2^46^ was applied to align Third-generation sequencing (TGS) data, Hifi reads, back to the assembly to assess its completeness and evenness of sequencing data. Statistic on TGS data alignment showed that of 1,913,134 total reads, 1,907,844 were mapped representing 99.72%. For gene prediction, embryophyta database in BUSCO^45^ containing 1,614 core genes was employed. Using BUSCO v4.0 to assess the integrity of gene prediction, 98.14% of BUSCO core genes were identified in predicted gene list, which indicates a high integrity.

**Table 6:** Statistics on NGS data alignment and Sequencing depth and coverage.

Total_reads	Mapped_reads	Mapped (%)	Properly_mapped_reads	Properly_mapped (%)
238,059,580	237,036,445	99.57	223,841,734	94.03
**Average depth**	**Coverage**	**Coverage(≥5X)**	**Coverage(≥10X)**	**Coverage(≥20X)**
49	99.92	99.71	99.28	95.83

## Data Availability

All software and pipelines were executed in accordance with the manuals and protocols provided by the published bioinformatics tools as described in the methods.
